# Examining Equity Factors in Healthy Aging Interventions: A Scoping Review of Healthcare Strategies

**DOI:** 10.1093/geroni/igaf122.2456

**Published:** 2025-12-31

**Authors:** Odelia Ben Harush, Chava Kurtz, Anna Zisberg, Stuart McLennan, Paola Buedo, Efrat Shadmi

**Affiliations:** University of Haifa, Haifa, HaZafon, Israel; University of Haifa, Haifa, HaZafon, Israel; University of Haifa, Haifa, HaZafon, Israel; Technical University of Munich, School of Medicine and Health, Munich, Bayern, Germany; Technical University of Munich, Munich, Bayern, Germany; University of Haifa, Haifa, HaZafon, Israel

## Abstract

Studies on interventions promoting healthy aging often do not address the effect of inequity on their implementation and outcomes. This scoping review aims to identify the equity-related factors explored in healthy aging strategies and interventions. A structured search of multiple electronic databases was conducted using keywords and indexed terms related to healthy aging interventions, with equity aspects including place of residence, race/ethnicity, occupation, religion, education, socioeconomic status, and social capital. A total of 5890 studies were identified and screened, of which 74 met the inclusion criteria. Approximately one-third of the studies (25, 34%) only described equity aspects in demographic characteristics but did analyze results according to these factors; Eleven studies (15%) adjusted for equity aspects; and 38 studies (51%) assessed outcomes by equity factors. Of the 38, 9 studies (24%) examined intervention effects while controlling for equity aspects, and 28 studies (74%) focused on interventions in minority or socioeconomically deprived groups. Of these 28 studies, 20 (71%) reported positive outcomes, primarily in quality of life and functioning. Only 9 studies (12%), explored multiple equity aspects (intersectionality). Equity-related aspects are rarely studied in research in health aging research. Studies examining interventions in minority or socioeconomically deprived groups generally show positive patient-reported outcomes, but none reported effects on healthcare resource use. Future research should prioritize analyzing outcomes by equity dimensions, especially intersectionality, to inform inclusive interventions that may reduce health inequities and promote healthy aging.

